# Effect of strain on thermoacoustic characteristics of lean premixed hydrogen flames

**DOI:** 10.1177/17568277251387948

**Published:** 2025-10-21

**Authors:** Emre Böncü, Nguyen A Khoa Doan, Ivan Langella

**Affiliations:** 1 Faculty of Aerospace Engineering, 2860 Delft University of Technology, Kluyverweg 1, HS Delft, The Netherlands

**Keywords:** Hydrogen, high strain, thermoacoustics, differential diffusion, computational fluid dynamics

## Abstract

Recent research [Porcarelli et al., *Int. J Hydrogen Energy* 49, 2024] shows NOx can be suppressed in lean premixed hydrogen flames at high levels of strain without loss of efficiency. However, the thermoacoustic response of such flames in high strain regimes remains unexplored. The present work addresses this by investigating the thermoacoustic characteristics of very lean premixed hydrogen flames under increasing levels of applied strain rates. High-fidelity simulations are conducted in a counterflow, reactants-to-products configuration, where strain rate is controlled by inlet velocities. Several simulations are conducted under a range of different strain rates and forcing frequencies. The simulations are run using a modified version of the reactingFOAM solver within OpenFOAM-9 that accounts for the different diffusivities in hydrogen flames and uses the low-Mach formulation of the Navier–Stokes equations. The forcing was imposed by applying sinusoidal oscillations on top of the uniform velocity of the reactants at the inlet boundary with a specific frequency and an amplitude of 10% of the mean value. Results indicate that above a threshold, increasing strain raises the gain and shifts its peak to higher frequencies. This behaviour stems from the flame speed response to strain at negative Markstein lengths and can be leveraged for the development of ultralow-emission combustion devices where applied strain can be used as an additional mechanism to shift an unstable flame response to different frequencies. Moreover, the highest applied strain rate investigated triggers an inversion of the Markstein length, leading to a 180 ∘ phase shift in the flame dynamic response. This aspect and further implications are discussed in the paper.

## Introduction

The world’s energy demand is currently met primarily by burning fossil fuels, a process that generates substantial CO ${}_{2}$ emissions. This dependency is unsustainable, as it significantly contributes to climate change, implying that a transition to cleaner energy sources is needed. Although energy sources such as solar, hydroelectric or wind energy are becoming increasingly significant for energy production as time progresses, combustion will remain an important energy source for humanity for the foreseeable future.^1^ Moreover, the level of technology of renewable energy sources for some sectors such as the aviation industry is not yet mature or limited to applications involving moderate power densities, implying that the share of combustion in the power generation sector will remain dominant in the foreseeable future. In this scenario, hydrogen is an attractive alternative to fossil fuels for the power generation and transportation sectors.^2^ Hydrogen is, in fact, carbon-free and can be produced cleanly using electrolysis via renewable energies. However, the use of hydrogen in combustion devices comes with challenges as a result of its high adiabatic flame temperature, implying that significant levels of toxic nitrogen oxides (NOx) can be produced. To reduce the emissions of NO ${}_{x}$ from hydrogen combustion, one can exploit the wide flammability limits of hydrogen flames and burn hydrogen under lean or ultra-lean premixed conditions, thus decreasing the adiabatic flame temperature and consequently the NOx via thermal route.^3–6^ However, the strong reactivity and diffusivity of hydrogen implies that challenges such as flashback or thermodiffusive instability can occur under very lean conditions,^7–10^ and these have to be fully understood before hydrogen can be employed safely in modern combustion devices.

Recently, Porcarelli et al.^11^ investigated the effect of strain on NO ${}_{x}$ emissions in lean premixed hydrogen flames, and it was found that at intensive applied strain regimes, NO ${}_{x}$ emissions are suppressed without efficiency loss due to a redistribution of OH radicals within the flame. This implies that NOx levels can be mitigated at not-so-lean conditions by the application of intensive strain levels, without thus incurring the risk of common instabilities found at ultra-lean conditions. However, the response of a lean premixed hydrogen flame subject to thermoacoustic perturbations is yet unknown at high strain regimes, and this information is of paramount importance to be able to design robust and safe combustion devices exploiting the strain-driven suppression of NOx. Thermoacoustic instabilities occur as a result of a constructive interaction between unsteady heat release and pressure fluctuations. These unwanted pressure fluctuations can reach amplitudes that can cause structural damage or adversely affect the combustion process, leading to issues such as flashback, flame blow-off, or quenching.^12^ The effect of strain on the thermoacoustic response was studied for counterflow diffusion hydrogen flames by Yao et al.^13^ numerically using 1D simulations, where it was found that increasing strain had the effect of lowering gain values at lower frequency range while increasing them for the higher frequency range. Furthermore, Yao et al. found that increasing strain had the effect of lowering the peak values of gain for counter-flow diffusion flames. The thermoacoustic response of premixed and non-premixed methane flames in counterflow were also analyzed by Zambon et al.,^14^ where it was observed that using a detailed chemical mechanism rather than a simple global one was important for the accuracy of thermoacoustic analyses. Finally, Tian et al.^15^ numerically analysed the flame transfer functions (FTFs) of conical and V-flames to determine the effect of the flame stretch imposed by the curvature of the flame, and found that increasing flame stretch had the effect of increasing gain in the high frequency range for premixed hydrogen flames. Nevertheless, despite these studies and to the best of the authors’ knowledge, no information exists yet on the thermoacoustic response of lean premixed hydrogen flames at moderate to high levels of applied strain. The present study tries to address this gap by shedding some light on the dynamic behaviour of premixed hydrogen flames at increasing strain levels.

The counterflow premixed flame configuration is a common way of analysing flame characteristics under strain. Although the flame thermoacoustic response is significantly affected by geometry and operative conditions in real scenarios, the relatively simple configuration chosen here serves to isolate the effect of strain on the flame response and is therefore chosen as first analysis in the present study. Both a symmetric reactant to reactant and an asymmetric reactant to exhaust configuration is possible for premixed counterflow flames. For this study, the asymmetric configuration is chosen to avoid possible flame-to-flame interactions at high strain rates in the symmetric configuration.^11^ Five different mean applied strain rates were investigated using high fidelity numerical simulations and examining a wide range of forcing frequencies. The velocity at the domain inlet at the reactants boundary was forced by superimposing oscillations in time for each specific frequency on the spatially uniform inlet velocity profile. The low-Mach assumption was used in the simulations to reduce computational cost and avoid the occurrence of possible numerical instabilities in the counterflow configurations due to the use of Navier–Stokes characteristic boundary conditions. Although the use of a low-Mach formulation for thermoacoustic analyses might be controversial, recent studies analysing the reliability of this formulation for thermoacoustic analysis^16^ have shown that for acoustically compact flames the differences between the fully compressible and low-Mach approaches are negligible when the forcing is applied within the linear regime.^16^ A flame is said to be acoustically compact when the heat release is concentrated in a region much smaller than acoustic wavelengths.^17^ The smallest wavelength used in the simulations is 17.2 cm which happens at 2000 Hz forcing. The average thickness for the simulated flames is 0.4 mm. Since all the flames considered here are all nearly planar and much thinner than the smallest wavelength considered in the study, they can be considered acoustically compact for all forcing frequency range, therefore the low-Mach assumption is retained for the analyses in the present study. In light of the above discussion, the objective of the present study is to shed light on the thermoacoustic response of lean premixed hydrogen flames under increasing levels of applied strain. The implication of differential diffusion in hydrogen flame is further discussed in terms of Markstein length L in order to shed light on its contribution to the flame response.

## Methodology

### Domain and problem description

**Figure 1.**
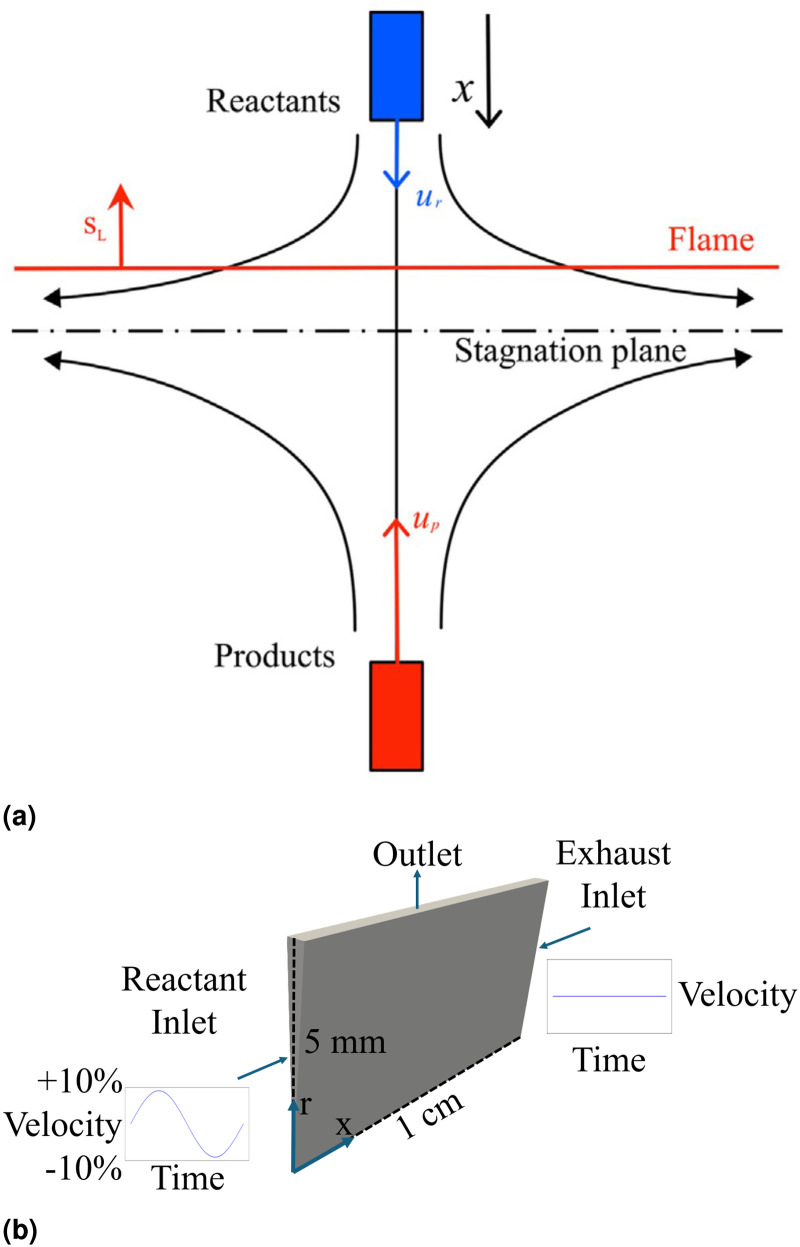
(a) Sketch of reactants-to-products (fresh-to-burnt) counter-flow, strained premixed flame configuration.^11^ (b) Sketch of the two-dimensional (2D) axisymmetric numerical domain used for the simulations in this study. Boundary conditions are also indicated.

The case studied in this work is the canonical reactants to products premixed flame configuration sketched in Figure 1(a). The reactant stream is set at an equivalence ratio of ϕ=0.4 . The hot gases stream consists of the products of the reactants at the same equivalence ratio, and its composition corresponds to that obtained assuming complete combustion for simplicity. Temperature is set to 300 K in the reactants and to 1425 K in the products, which corresponds to the adiabatic flame temperature of the freely-propagating flame under equilibrium conditions. Pressure is set to 1 atmosphere for all cases investigated.

The simulations for the study are conducted in a two-dimensional (2D) axisymmetric domain with a length L= 1 cm between the exhaust and reactant inlets, a radius of 5 mm, and a 2.5 ∘ angle between the two side faces. Spatially uniform velocity profiles are imposed at both inlets, and temporal variations are introduced on the reactant side by superimposing oscillations in time with an oscillation amplitude of 10% of the mean velocity. Simulations are performed with forcing frequencies between 50 and 2000 Hz. The domain and velocity boundary conditions are illustrated in Figure 1(b). Five different applied mean strain rates are investigated, respectively 500, 1000, 1500 , 2000 and 5000 $s^{\text{-}}1$ , to determine the effect of strain on the thermoacoustic response. The mean applied strain rate a used in this study is defined as

(1) $$a=\frac{\left|u_{r}-u_{ex}\right|}{L}$$

where $u_{r}$ and $u_{ex}$ are respectively the velocity imposed at the reactants and product sides. For each applied strain rate 26 different frequencies are simulated, corresponding to 26×5=130 simulations in total. Forcing was introduced after an initial transient to obtain an (unforced) steady state solution, and the simulations was run for 5 oscillation periods.

The streamwise and radial directions are uniformly meshed using 500 and 200 cells, respectively, corresponding to about 20 cells in the unstretched laminar flame thickness. A mesh sensitivity analysis was performed on a subset of the frequency range. Specifically, a finer mesh with 400 and 1000 uniformly spaced cells in the radial and streamwise direction was used for comparison. The results obtained for both meshes will be presented in the Results section.

### Governing equations

The reacting Navier–Stokes equations are solved using the finite volume methodology and a modified *in house* version of the reactingFOAM solver within OpenFOAM v9. The low-Mach formulation is used to decouple thermodynamic pressure and velocity field under the assumption that the flame is acoustically compact.^16^ The resulting set of governing equations consists of continuity, momentum, species and specific sensible energy equations as shown below:

(2) $$\frac{\partial \rho }{\partial t}+\nabla \cdot (\rho v)=0$$

(3) $$\frac{\partial (\rho v)}{\partial t}+\nabla \cdot (\rho vv)-\nabla \cdot \bar{\bar{\tau }}=-\nabla p$$

(4) $$\frac{\partial \rho Y_{i}}{\partial t}+\nabla \cdot (\rho Y_{i}v)+\nabla \cdot (\rho Y_{i}V_{i})=w_{i}$$

(5) $$\frac{\partial (\rho h)}{\partial t}+\nabla \cdot (\rho vh)=-\nabla \cdot q+\dot{Q}$$

In the above equations v is the velocity vector, p is the pressure, $Y_{i}$ is the mass fraction of the i th species, $V_{i}$ is the diffusion velocity of the i th species, $w_{i}$ is the mass reaction rate of i th species, h is the specific sensible enthalpy of the mixture, $\bar{\bar{\tau }}$ is the viscous stress tensor, q is the heat flux vector and $\dot{Q}$ is the heat release rate from the chemical reactions. According to Stokes hypothesis, $\bar{\bar{\tau }}$ is defined as

(6) $$\bar{\bar{\tau }}=-\frac{2}{3}\mu (\nabla \cdot v)\bar{\bar{I}}+\mu \left[\nabla v+(\nabla \;v{)}^{T}\right]$$

where μ is the dynamic viscosity and $\bar{\bar{I}}$ is the identity tensor. The flux vector q is defined as

(7) $$q=-\lambda \nabla T+\rho \sum_{i=1}^{N}h_{i}Y_{i}V_{i}$$

where λ is the thermal conductivity of the mixture, T is the temperature, $h_{i}$ is the specific enthalpy of the i th species and N is the number of species.

Differential diffusion is taken into account by using a mixture averaged model^18^ for the diffusion velocities, where Soret effect was ignored for simplicity and its effect will be assessed in future studies. $V_{i}$ is thus given by

(8) $$V_{i}=-\frac{D_{i,\text{mix}}}{X_{i}}\nabla X_{i}+V_{c,i}$$

where $D_{i,\text{mix}}$ is the mixture-averaged diffusion coefficient of the i th species, which is calculated in the solver from the binary diffusion coefficients of the species $D_{i,j}$ as

(9) $$D_{i,\text{mix}}=\frac{1-Y_{i}}{\sum_{\;j\neq i}^{N}\frac{X_{i}}{D_{ij}}}$$

where $X_{i}$ is the molar mass of species i . The diffusion velocity for each species is further corrected to ensure the continuity requirement $\sum_{k=1}^{N}j_{k}=1$ , where $j_{k}$ are the species mass fluxes. This results in adding a velocity correction $V_{c,i}=\sum_{k=1}^{N}D_{k}^{M}Y_{k}$ to Equation (8). The approximation of Hirschfelder et al.^19^ is used to compute the binary diffusion coefficients $D_{ij}$ .

The Conaire et al. mechanism^20^ is used as chemical kinetics mechanism. The reaction rate constants for each reaction are calculated using the Arrhenius equation:

(10) $$k=AT^{b}\exp\;\left(-\frac{E_{a}}{RT}\right)$$

where A is the pre-exponential factor, $E_{a}$ is the activation energy, b is the temperature power exponent and R is the universal gas constant. Temperature is computed using the specific sensible enthalpy and the specific heat of the mixture, which in turn is computed using the mass-weighted average of specific capacities obtained from the JANAF polynomials. The mixture viscosity is calculated a priori according to the procedure described in Wilke^21^ Evlampiev.^22^ Finally, density is computed via the ideal gas equation of state using temperature and operative pressure, $p_{\mathrm{ref}}=1$ atm, consistently with the low-Mach formulation.

### Numerical details

The pressure implicit with splitting operator^23^ method was used to couple pressure and velocity in the Navier–Stokes equations, where the Poisson type pressure equation is solved within this loop to retrieve the pressure field in the momentum equation. Third order cubic Gauss schemes are used for the convective terms in all transport equations. The Crank-Nicolson scheme is used for the time derivative. An adaptive time-stepping approach was used to keep the maximum Courant number below 0.4 at each time step. Thermodynamic and transport properties of species were determined by using polynomials fitted over the range of temperatures relevant for the current study.

### Post processing

To determine the flame response to reactant inlet velocity oscillations, the area integral of the heat release rate ( $\dot{Q}$ ) field is calculated for each time step, resulting in a time series signal. This signal is normalised at the end of each simulation by its mean, and the Discrete Fourier transform (DFT) of the normalised area integral $\dot{Q}$ versus time is then calculated along with the power density at the specific forcing frequency simulated. The gain value is calculated by dividing the power density result of the normalised area integral $\dot{Q}$ versus time by the power density of the normalised reactants inlet velocity. The gain G and phase difference φ are then defined as:

(11) $$G=\frac{\left|\hat{\dot{Q}}(f)\right|}{\left|\hat{u}(f)\right|}$$

(12) $$\varphi =\arg\;\left(\hat{\dot{Q}}(f)\right)-\arg\;\left(\hat{u}(f)\right)$$

where $\left|\hat{\dot{Q}}(f)\right|$ and $\left|\hat{u}(f)\right|$ are respectively the magnitude of the DFT of $\dot{Q}$ and the magnitude of the DFT of the reactants inlet velocity at the forcing frequency f , while $\arg\;(\hat{\dot{Q}}(f))$ and $\arg\;(\hat{u}(f))$ are respectively the phases of the DFT of $\dot{Q}$ and of the reactant inlet velocity at the same forcing frequency. The phase difference between the two signals is calculated by subtracting the phase of the normalised reactant inlet velocity in time from the phase of the normalised area integral $\dot{Q}$ at the same forcing frequency. Since a non-uniform time stepping scheme was used in the simulations, the time series were resampled using linear interpolation to make the time steps between the values uniform.

## Results and discussion

### Overall flame characteristics

**Figure 2.**
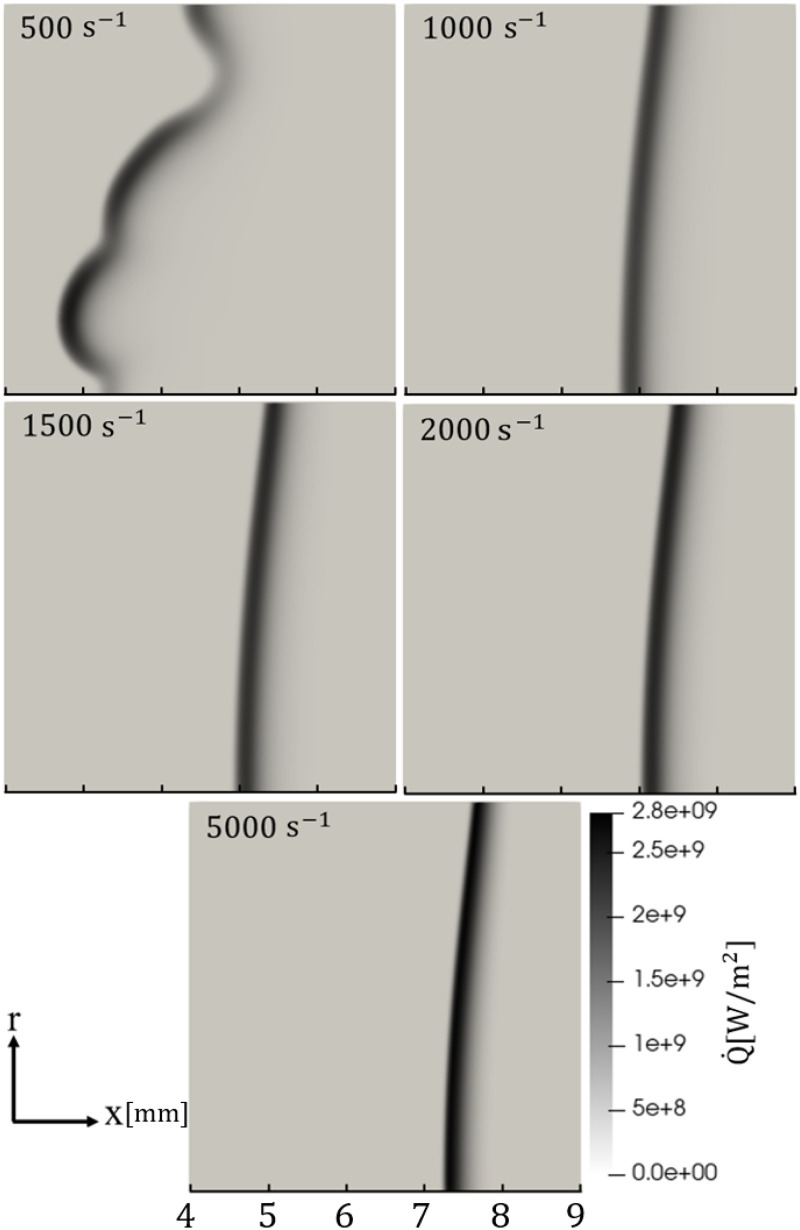
Heat release rate, $\dot{Q}$ , contours of the unforced lean premixed hydrogen flame in reactants to products counterflow configuration for various levels of applied strain level, a : thermodiffusively unstable case ( $a=500\;{\text{s}}^{-1}$ ) and stable cases ( $a=1000\;{\text{s}}^{-1}$ to $a=5000\;{\text{s}}^{-1}$ ). Reactants enter the domain from the left. The ticks in the x direction are spaced by 1 mm.

The hydrogen strained flame response under thermoacoustic forcing is investigated in this section. For all strain rates except 500 s^−1^ the flame before the imposition of oscillations at the boundary was observed to be steady, although not perfectly flat due to the effect of the boundary conditions, which slightly affect the axial velocity field near the top exit boundary. For the 500 s^−1^ strain rate case, the occurrence of thermodiffusive instabilities was observed. To show this, contours of heat release rate ( $\dot{Q}$ ) field for all of the strain rates under constant inlet velocity are shown in Figure 2. The thermodiffusively unstable case is shown for a random representative time, while all other cases are shown at the steady state, after a small transient is passed. It is interesting to note that thermodiffusive instabilities are somewhat suppressed as strain rate increases. This stabilizing effect of strain was also observed by Sivashinsky et al.^24^ under varying amount of strain rate. Since the fluctuations resulting from the thermodiffusive instability overpower the oscillations imposed to the reactant inlet velocity in the cases with forcing, the 500 s^−1^ strain results are not further analysed here.

### Mesh sensitivity

**Figure 3.**
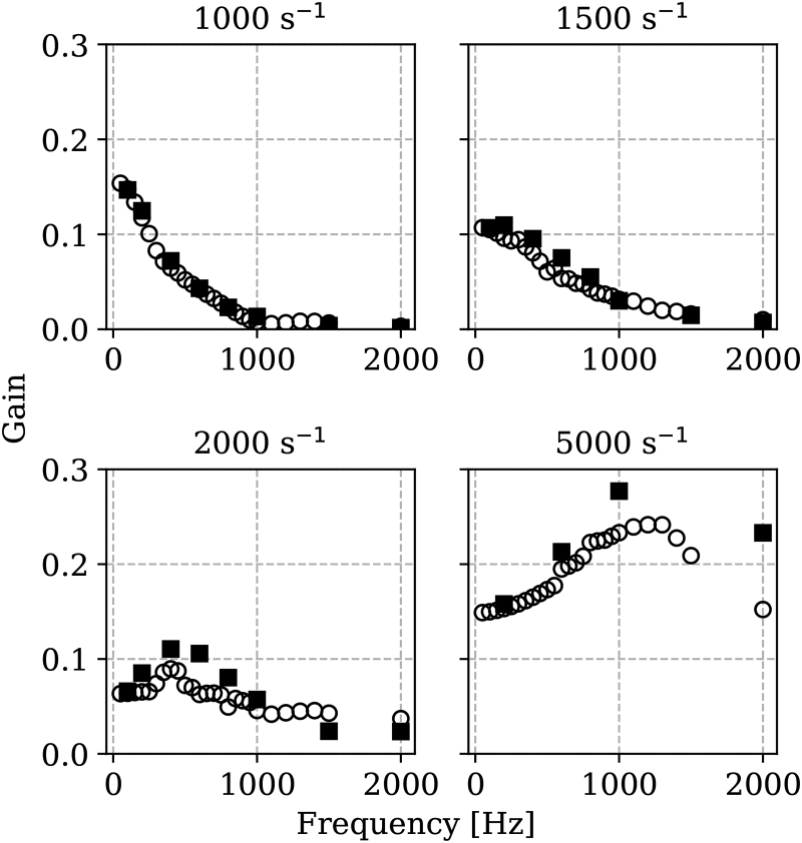
Gain of heat release rate fluctuations as function of imposed frequency of velocity oscillation at the reactants boundary for different strain rates, obtained using the coarser mesh of 100,000 elements ( ◯ ) and the finer mesh of 400,000 elements ( ■ ).

**Figure 4.**
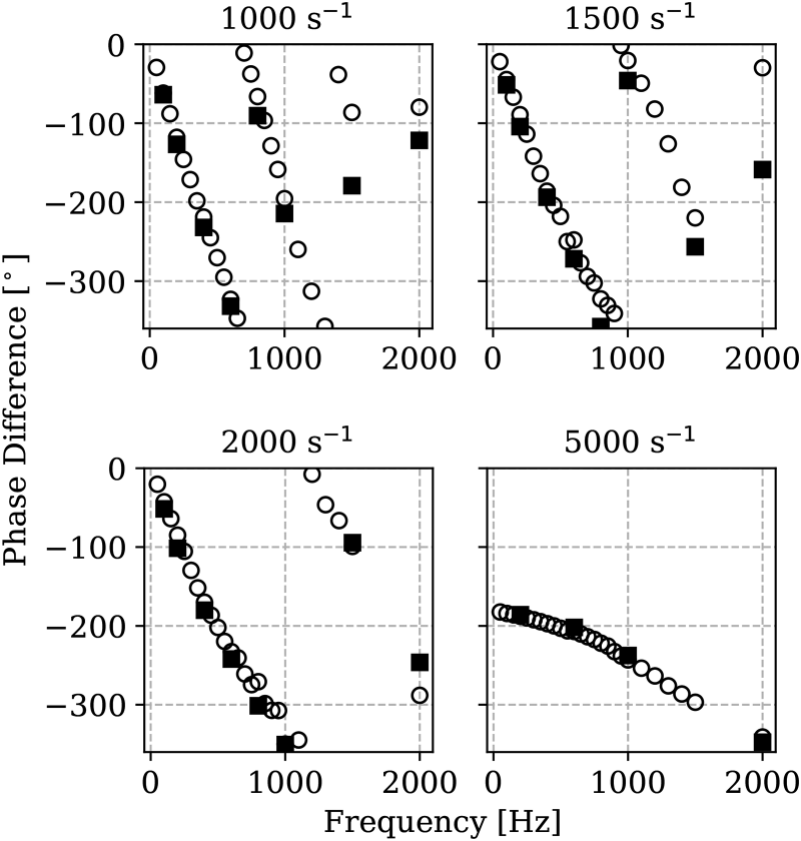
Phase difference of heat release rate fluctuations as function of imposed frequency of velocity oscillation at the reactants boundary for different strain rates, obtained using the coarser mesh of 100,000 elements ( ◯ ) and the finer mesh of 400,000 elements ( ■ ).

The dynamic response of the flame for the cases at $a\geq 1000\;{\text{s}}^{-1}$ is analysed next. Gain G and phase difference φ for different applied strain rates are shown as function of the forcing frequency f in Figures 3 and 4 respectively. Results obtained for the two meshes described in the Methodology section are shown for comparative purposes. The gains as reported here are not normalised to approach unity at zero forcing frequency. First of all, the figures indicate that the coarser mesh is already sufficiently accurate to capture the flame dynamic response at low and moderate strain rates. For the strain rate of 2000 s^−1^ some underestimation is observed for the coarser mesh in terms of gain amplitude in the region of the peak. Similarly for the 5000 s^−1^ some underestimation at the higher frequency range is observed for the coarser mesh. Nevertheless, the location of the peaks and the overall trend are still well captured by the coarser mesh. In terms of phase shift, no significant differences are observed in the results obtained using the two meshes independently of the applied strain. However, some overestimation of phase shift is observed for the coarser mesh as compared to the finer mesh for frequencies above 1500 Hz. Since the flame thickness in the case of hydrogen premixed flames does not vary significantly with strain in the range investigated here, the decrease of accuracy as strain rate or imposed oscillation frequency increase is imputable to the capturing of the velocity gradients. As the overall trend is well captured by the coarser mesh, this mesh is retained for the following analyses.

### Analysis of gain and phase shift

**Figure 5.**
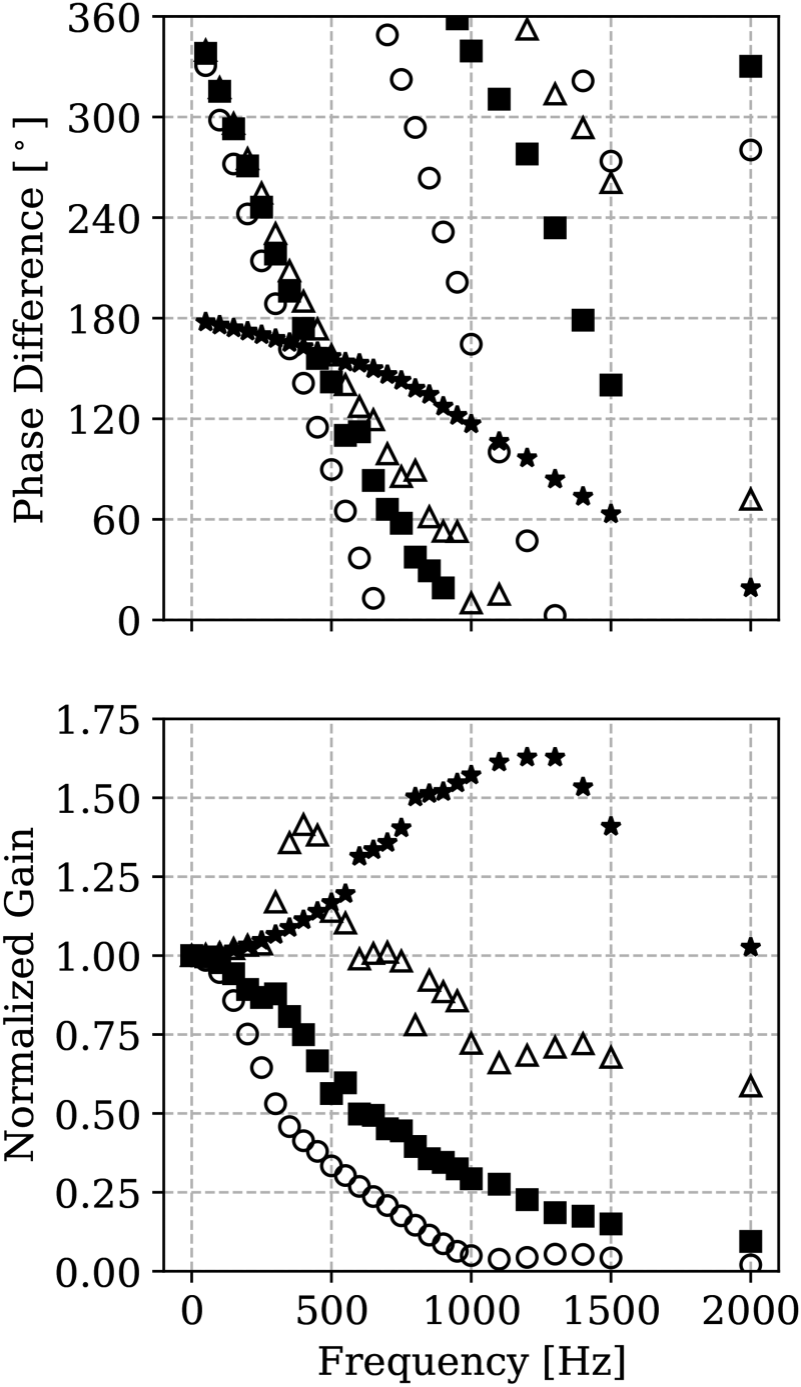
Phase difference (top) and normalised gain (bottom) of heat release rate fluctuations as function of imposed frequency of velocity oscillation at the reactants boundary for different applied strain rates: 1000 s^−1^ ( ◯ ), 1500 s^−1^ ( ■ ), 2000 s^−1^ ( △ ) and 5000 s^−1^ ( ⋆ ).

Results in terms of gain and phase shift for the coarser mesh for all thermodiffusively stable cases are plotted again in Figure 5, where the gain is normalised to approach unity as the frequency goes to zero for clarity of discussion. Since the forcing amplitude increases with strain, in order to normalise the gain values with the correct forcing amplitude two additional simulations were performed for all strain rates, where the velocity at the reactants inlet was linearly ramped up and down respectively (without superimposing any further oscillation). The resulting $\dot{Q}$ signal was consequently observed to linearly increase or decrease. The gain at zero frequency was thus calculated by dividing the slope in time of the integrated heat release rate signal by the slope in time of the reactants inlet velocity. This method of normalising also accounts for the fact that part of the fuel exits the domain in different amounts for different applied strain rates in the studied configuration. These results for gain and phase shift are discussed below.

#### Gain

By looking at the results in terms of gain in Figure 5, one can notice that for applied strain rates up to 1500 s^−1^ the gain value always decreases, implying a thermoacoustically stable condition. This decrease is steeper for the case at lower strain. As one moves to the higher strains of 2000 and 5000 s^−1^, a peak in gain G>1 appears. The amplitude of this peak further seems to grow with strain and shift towards higher frequencies, which might be the result of the higher fuel flow rate (thus higher power) at higher levels of strain. In light of this, the results seem consistent with the results obtained for example for a different configuration in Barbosa et al.,^5^ Oztarlik et al.,^25^ where the addition of hydrogen was observed to shift the thermoacoustic unstable response towards higher powers. The results presented here are also consistent with those reported by Tian et al.,^15^ where it was shown that with increasing flame stretch the gain values increased in the higher frequency range. In their paper, Tian et al. also noted that there are two main mechanisms for which the flame stretch affects the FTF. The first is the stabilizing effect of strain, where increasing the strain can decrease the wrinkling of the flame, resulting in damping of gain values for low frequencies. The second mechanism is associated to the fact that strain affects the consumption speed of the flame depending on the value of the Markstein length. The Markstein length represents the effect of flame stretch on the consumption speed of the flame. Two factors contribute to the total flame stretch, strain rate and flame curvature. The effect of curvature was investigated in a previous study,^26^where it was shown that the effect of flame stretch created by the curvature of the flame is important for the prediction of the laminar flame speed for high curvature flames like spherical flames. Nevertheless, since in our simulations the flames are mostly flat and under intensive strain rate, only the flame stretch created by strain is taken into consideration here, and consequently the Markstein length used in the following analysis is to be considered only related to flame strain. The Markstein length for the present simulations at ϕ=0.4 is negative, implying that strain increases the flame speed. It is worth noting that in the work of Tian et al. only curvature was used to impose the effect of stretch on the flame, while strain is studied in the present study. Also, the range of stretch rates investigated in the present work (in terms of strain) is significantly wider than that explored in Tian et al.^15^ Since the Markstein number is known to impact the flame propagation differently depending on whether curvature or strain is the driving mechanism behind the overall stretch,^27^the results presented above provide further critical details on the effect of strain on the FTF in premixed hydrogen flames.

#### Phase difference

The phase difference results reported in Figure 5 are discussed next. For the strain rate levels of 1000, 1500 and 2000 s^−1^, the phase difference starts from values of φ≈0 as one would expect. As the frequency increases the phase lag between forcing and flame response also increases. However, it is interesting to note that the rate of increase (in absolute value) of the phase lag is less steep as strain increases, suggesting that the flame response is becoming faster. This effect is due in part to the fact that the movement of the flame in the streamwise axial direction with increasing strain is limited as strain increases and the flame gets closer to the stagnation plane. This implies that the flame cannot freely counteract the action of the oscillating impinging velocity by adjusting its position in space. Further considerations apply instead for the case of applied strain rate of $a=5000\;{\text{s}}^{-1}$ , also shown in Figure 5. In this case in fact the phase difference at f=0 Hz is observed to start at a phase value of $\varphi ={-180}^{\circ }$ . This behaviour implies that the further increases in the reactants velocity from the conditions at lower applied strain discussed earlier cause the volume integral of the reaction rate $\dot{Q}$ to decrease. The reason for this is that at $a=5000{\text{s}}^{-1}$ the Markstein length L , or equivalently the Markstein number $\mathrm{Ma}=L/\delta_{\mathrm{th}}$ ( $\delta_{th}$ being the laminar flame thickness) has inverted from negative to positive, and thus, increases of applied strain rates (due to increasing velocities of the reactants) yield a decrease of flame speed and vice versa.

**Figure 6.**
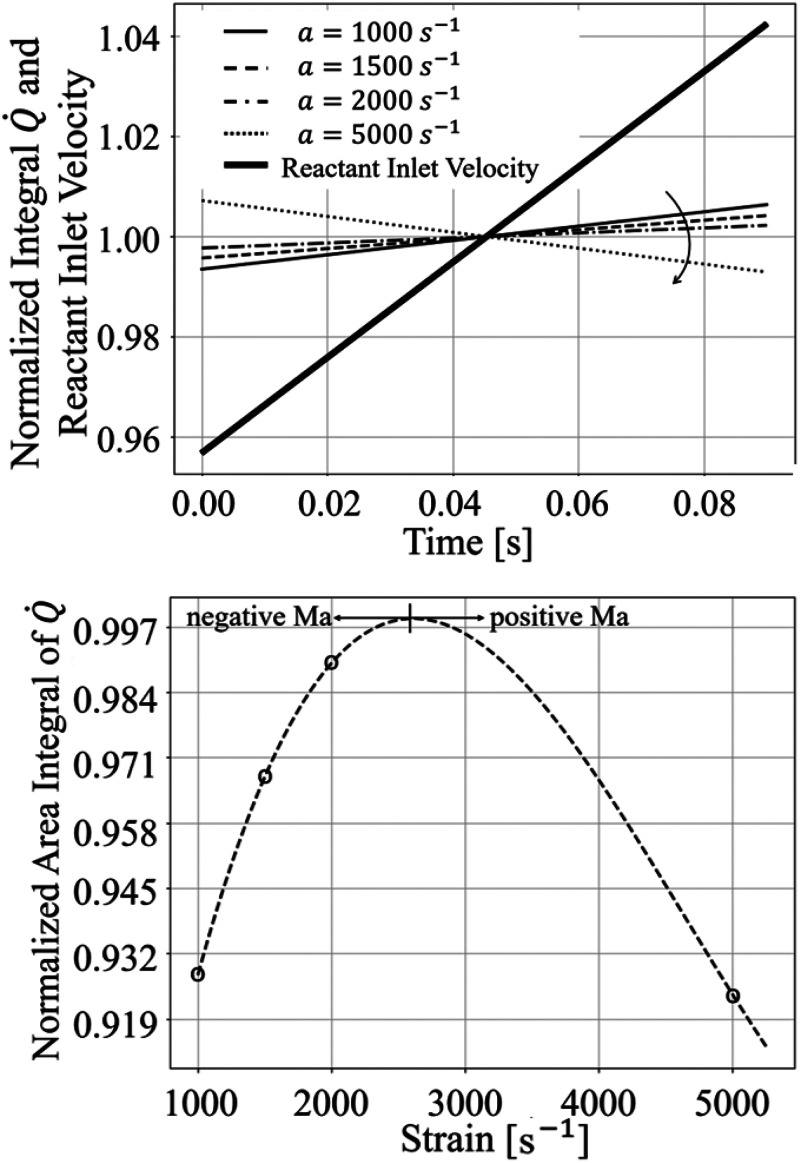
Variation in time of normalised integral heat release rate for increasing levels of applied strain and for a linear imposed increase of velocity at the reactants inlet (top). Normalised area integral of heat release rate versus applied strain (bottom).

Figure 6 (bottom) shows the effect on the volume-integrated heat release rate of an increase in time of velocity at the reactants inlet. As one can notice, the slope of this increase flattens as strain increases (see direction of the arrow in the figure), until changing sign. It is worth noting that the behaviour at $a=5000\;{\text{s}}^{-1}$ is still linear, suggesting that the flame is not approaching extinction or losing in efficiency. Indeed, the aforementioned behaviour is related to the Markstein number sign, which can be further proven by looking at the top graph in Figure 6. Here the behaviour with applied strain rate a of the normalised volume-integrated reaction rate, which is proportional to the flame consumption speed, is shown. The curve is obtained by interpolating the data associated to four computed nominal applied strain values using a third order polynomial. As one can notice, at the very lean equivalence ratio of ϕ=0.4 investigated here, the Markstein number inverts its sign at about $a\approx 2500\;{\text{s}}^{-1}$ (in the limit of accuracy of the polynomial fit). On the other hand, the value of normalised heat release rate at $a=5000\;{\text{s}}^{-1}$ is comparable to that at $a=1000\;{\text{s}}^{-1}$ (which is above the value for an unstretched flame), suggesting that the flame is not yet approaching extinction. This results in the phase shift of 180 ∘ at the highest strain rate observed in Figure 5 as compared to the lower strain rates investigated. To the best of the authors’ knowledge, this is the first time such a behaviour is observed, and it can only occur for very lean flames with negative Lewis number (implying a negative Ma at unstretched conditions) when relatively high levels of strain rates are imposed.

As the forcing frequency f is increased, the phase difference in Figure 5 for the case at $a=5000\;{\text{s}}^{-1}$ increases in absolute value as expected, but at a less steep rate than for the other applied strain rate cases, which is consistent with the trends observed for the lower strain regimes. These results indicate that, although the investigation conducted here is for a simple counterflow flame and quantitative results might change according to the specific configuration used in a real engine, applied strain can be used to alter the thermoacoustic response of a hydrogen flame in combustion system devices by altering the phase lag and, as discussed earlier, the frequency range at which the gain is positive.

## Conclusion

In this study, the thermoacoustic response of lean premixed hydrogen flames under increasing strain rates was analysed for a reactants-to-products counterflow configuration. Detailed chemistry, high fidelity numerical simulations were performed with a modified version of the reactingFOAM solver in OpenFOAM to account for differential diffusion effects at lean conditions. The flames were forced over a range of frequencies and strain rates, and the resulting gain and phase responses were investigated.

The results showed that the strain rate significantly influences the FTF, particularly at higher frequencies. In particular, the following scenarios are found:

- At low to moderate strain rates ( $a\approx 500\;{\text{s}}^{-1}$ ) thermodiffusive instabilities dominate the flame dynamic behaviour;
- At moderate applied strain rates ( $1000\;{\text{s}}^{-1}<$ $a<2000\;{\text{s}}^{-1}$ ), thermodiffusive instabilities are suppressed by strain and the gain of thermoacoustics oscillation is below unity, implying these flames are stable;
- For high strain rates ( a> 2000 s^−1^) the gain of thermoacoustics instabilities increases with strain in amplitude and shifts towards higher frequencies. This behaviour is associated with an increase in the consumption speed with strain due to the negative Markstein length for the configuration studied.

In terms of phase lag between heat release rate and forced velocity, results indicate that while this phase lag increases in magnitude as expected as the forcing frequency f increases, applied strain limits this rate of increase. Moreover, for the highest strain rate an inversion of the Markstein length from negative to positive is observed, leading to a phase shift of 180° in the flame dynamic response as compared to the cases at lower strain rate. The results presented in the present work reinforce the idea that strain has a destabilizing effect on thermoacoustics behaviour at higher frequencies. On the other hand, results also indicate that the thermoacoustic response can be changed by changing the applied strain rate on the flame. This can have important implication for the realisation of modern and safe combustion devices, where a thermoacoustically unstable condition can be avoided by shifting the frequencies at which thermoacoustic instabilities occur by varying the level of applied strain. This could be for example the case of a bluff body^28^ where the level of tangential strain on the flame can be increased by increasing the inlet speed, as this affects the shear layer where the flame stabilises. This will be the object of future work. Same effect can be achieved in practical burners such as swirl burners by adjusting the swirl number or confinement, and in multi-jet arrays by modifying jet spacing or co-flow, each primarily acting through their impact on local strain.
